# Pharmacokinetics Studies of 12 Alkaloids in Rat Plasma after Oral Administration of Zuojin and Fan-Zuojin Formulas

**DOI:** 10.3390/molecules22020214

**Published:** 2017-01-30

**Authors:** Ping Qian, You-Bo Zhang, Yan-Fang Yang, Wei Xu, Xiu-Wei Yang

**Affiliations:** 1 State Key Laboratory of Natural and Biomimetic Drugs, Department of Natural Medicines, School of Pharmaceutical Sciences, Peking University Health Science Center, Peking University, No. 38, Xueyuan Road, Haidian District, Beijing 100191, China; qianp@bjmu.edu.cn (P.Q.); zybo5288@163.com (Y.-B.Z.); yangyanfang@bjmu.edu.cn (Y.-F.Y.); high-xu@163.com (W.X.); 2 Department of Pharmacognosy, School of Pharmacy, China Medical University, Shenyang 110122, China

**Keywords:** Zuojin formula, Fan-Zuojin formula, Coptidis Rhizoma, Euodiae Fructus, alkaloids, pharmacokinetic

## Abstract

Zuojin formula (ZJ) is a traditional Chinese medicine (TCM) prescription consisted of Coptidis Rhizoma (CR) and Euodiae Fructus (EF), and has been used to treat gastrointestinal (GI) disease for more than 700 years. Fan-Zuojin formula (FZJ) is a related TCM prescription also consisted of CR and EF with the opposite proportion. In recent years, ZJ was getting more attention for its antitumor potential, but the indeterminate pharmacokinetic (PK) behavior restricted its clinical applications, and the PK differences between ZJ and FZJ were also largely unknown. Consequently it is necessary to carry out a full-scale PK study to demonstrate the physiological disposition of ZJ, as well as the comparative PK study between ZJ and FZJ to illustrate the compatibility dose effects. Therefore a liquid chromatographic–tandem mass spectrometry (LC–MS/MS) method was established and validated for the determinations of coptisine, epiberberine, palmatine, berberine, 8-oxocoptisine, 8-oxoepiberberine, noroxyhydrastinine, corydaldine, dehydroevodiamine, evodiamine, wuchuyuamide-I, and evocarpine in rat plasma. PK characteristics of 12 alkaloids after oral administration of ZJ and FZJ were compared, and the result was analyzed and discussed with the help of an in silico study. Then an integrated PK study was carried out with the AUC-based weighting method and the total drug concentration method. The established method has been successfully applied to reveal the PK profiles of the 12 alkaloids in rat plasma after oral administration of ZJ and FZJ. The results showed that: (1) double peaks were observed in the plasma concentration-time (C–T) curves of the alkaloids after ZJ administration; but the C–T curves approximately matched the two-compartment model after FZJ administration; (2) There were wide variations in the absorption levels of these alkaloids; and even for a certain alkaloid, the dose modified systemic exposure levels and elimination rate also varied significantly after administration of ZJ and FZJ extracts. The results could be interpreted as follows: firstly, inhibition effect on GI motility caused by the high content CR alkaloids (especially berberine) in ZJ could delay the T_max_, and increase the absorption and systemic exposure levels of the other alkaloids, and also lead to the double peak phenomenon of these alkaloids. However, for quaternary protoberberine alkaloids (QPA), double peaks were primarily caused by the different *K*a value in two intestinal absorption sites; Secondly, absorption was the major obstacle to the systemic exposure level of the alkaloids from CR and EF. In silico and PK studies suggested that the absorption of these alkaloids, except QPAs, mainly depended on their solubility rather than permeability; Thirdly, EF could promote the absorption and accelerate the elimination of QPAs, and had a greater influence on the former than the latter. At last the integrated PK analysis suggested that berberine and dehydroevodiamine could be regarded as the representative components to reflect the PK behaviors of CR and EF alkaloids after administration of ZJ and FZJ. In conclusion, the absorption, elimination and systemic exposure level of these alkaloids were mainly influenced by the proportion of EF and CR, the pharmacological effect on GI motility, and the physicochemical property of these alkaloids. These findings would be helpful for a better understanding of the activities and clinical applications of ZJ, FZJ and other related TCM prescriptions.

## 1. Introduction

Zuojin formula (ZJ) is a traditional Chinese medicine (TCM) prescription consisted of Coptidis Rhizoma (CR, the rhizome of *Coptis chinensis* Franch.) and Euodiae Fructus (EF, the fruit of *Euodia rutaecarpa* (Juss.) Benth.) at a ratio of 6:1. ZJ has been used to treat gastrointestinal (GI) disease for more than 700 years in China. In modern clinics, it is mainly used to treat gastric ulcer, gastroesophageal reflux disease, gastritis, pyloric obstruction, etc. [1]. In recent years, ZJ has been investigated for its antitumor activities [2,3,4,5], but the ambiguous pharmacokinetic (PK) study restricted its clinical applications. The PK studies of three quaternary protoberberine alkaloids (QPAs), berberine, palmatine and jatrorrhizine in rat plasma after administration of the CR–EF powder had already been reported [6], Our previous studies had discovered that compounds isolated from ZJ had multiple structural skeletons except QPAs [7,8,9], Consequently, it is necessary to carry out a multi-components PK study of ZJ, and both structure and bioactivity should be concerned in the selecting of quantitative markers.

**Figure 1:**
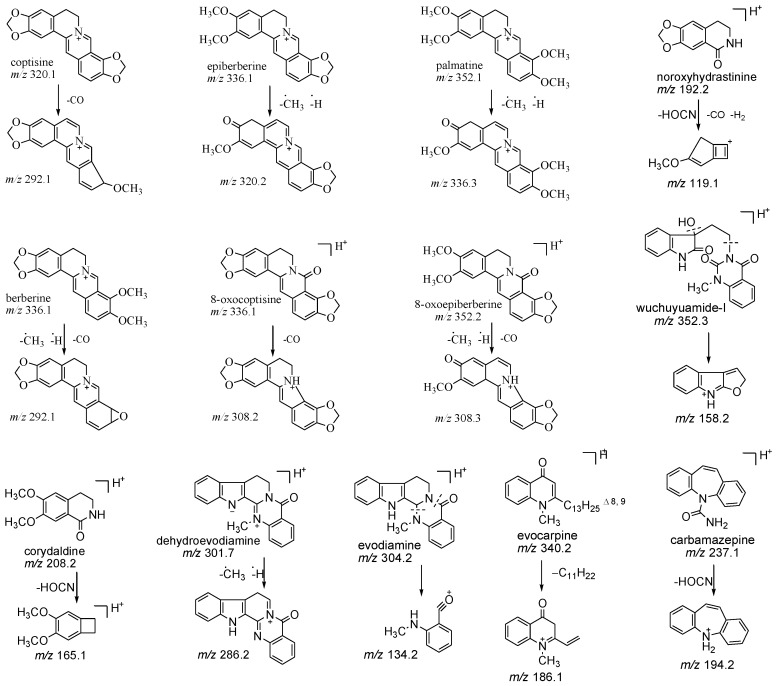
Chemical structures of the precursor and product ions of the 12 alkaloids and carbamazepine (internal standard, IS).

Alkaloids were the major components both in CR and EF. Berberine, palmatine, coptisine and epiberberine, all belonging to QPAs, were the most abundant alkaloids in CR [10], Studies demonstrated that they had wide spectrum of pharmacological effects, including anti-diarrheal [11], anti-inflammatory [12], anti-cancer [13,14] activities, and therapeutic potential on diabetes, hyperlipemia, hypertension [15], cardiovascular system [16,17] and central nervous system [18,19] diseases. Besides QPAs, tertiary protoberberine alkaloid (TPA) and simple isoquinoline alkaloid (SIA) were other two types of alkaloid in CR, and the major components were 8-oxocoptisine, 8-oxoepiberberine, noroxyhydrastinine and corydaldine. TPAs and SIAs also had notable bioactivities. For example, 8-oxocoptisine had gastric mucous membrane-protective activity [20], and could inhibit *P*-glycoprotein (*P*-gp) mediated multidrug resistance [21]. Therefore four QPAs, two TPAs and two SIAs from CR were selected as the quantitative markers in the PK study. Evodiamine and dehydroevodiamine, belonging to indoloquinazoline alkaloids (IQAs) with five unbroken ring structures, were two major alkaloids in EF, and had been reported to have anti-inflammatory [22], anti-cancer [23], neuroprotective [24,25], and vasodilatory [26] activities. EF also contained IQAs with broken ring structures [7], and wuchuyuamide-I was chosen as one of the quantitative markers to reflect the PK behavior of this type of alkaloid. Besides IQAs, EF also contained quinolone alkaloids (QLAs), which had anti-bacterial [27], anti-inflammatory [28] and anti-cancer [29] activities. Since evocarpine was one of the most abundant QLAs in EF [30], it should also be monitored in the PK study. Chemical structures of the selected 12 alkaloids were shown in Figure 1.

In previous PK studies of ZJ and related TCM prescriptions, discussions about the simple PK results were insufficient, such as the lacked unambiguous interpretation about the double peaks observed on plasma drug concentration-time (C–T) curves. Fan-Zuojin formula (FZJ) was a TCM prescription also consisted of CR and EF, but with the opposite proportion (1:6) [4]; accordingly, a comparative PK study of ZJ and FZJ would be helpful to reflect how the proportion of EF and CR influenced the PK behaviors of the alkaloids from the two herbs. As the absorption level of compounds mainly influenced by their solubility and permeability [31,32], an in silico assessment was necessary to interpret the wide variations of the absorption and system exposure level between the alkaloids with different structural skeletons. At last, the integrated PK study could give a more visualized C–T curves and PK parameters of CR and EF alkaloids. Area under curve (AUC)-based weighting method was the most frequently used integrated method in the TCM PK study [33,34,35], whereas the total drug concentration method was more concise, thus the results calculated by the two methods deserved to be compared and discussed. Taking into consideration the previous issues, the main goals of the present research was to develop a reliable liquid chromatographic–tandem mass spectrometry (LC–MS/MS) method to analyze the rat plasma pharmacokinetics of the main alkaloids from CR and EF, as well as to provide useful information for better understanding the physiological disposition, pharmacological effect and clinical application of ZJ, FZJ and other related TCM prescriptions.

## 2. Results and Discussion

### 2.1. Optimization of Chromatographic and Mass Conditions

Mobile phase was optimized by comparing acetonitrile (ACN)–water (H_2_O) and methanol (MeOH)–H_2_O solvent systems. The mobile phase consisted of ACN and H_2_O was confirmed finally with results of higher response, lower background noise and shorter analysis time than that of MeOH–H_2_O. Moreover, the addition of a buffer system consisted of 0.1% formic acid (*v*/*v*) and 11 mmol/L ammonium formate could improve the peak symmetry, ionization effect and sensitivity of the analytes.

All the tested alkaloids and internal standard (IS) all had better responses in positive mode than negative mode in ESI optimization. The Q1 and Q3 of the 12 alkaloids and IS were detected by infusing the standard solutions (200 ng/mL) in scan and product ions mode, respectively (structures see Figure 1). Key parameters, such as DP, CE and CXP were also optimized.

### 2.2. Sample Preparation

Different solvents, such as MeOH, MeOH–chloroform (3:1), and MeOH–ACN (2:1) (*v*/*v*), were used as protein precipitation reagents, and the extraction efficiencies were compared. The results showed that MeOH led to extremely low recoveries for all analytes, and MeOH–ACN (2:1) was better than MeOH–chloroform (3:1) because of the lower background noise. Liquid-liquid extraction method was also evaluated but finally not adopted, because the polarities of the analytes varied widely and it was difficult to find an appropriate solvent to extract all analytes well. Thus, plasma samples were treated with MeOH–ACN (2:1) as described in Section 3.6.

### 2.3. Method Validation

**Figure 2:**
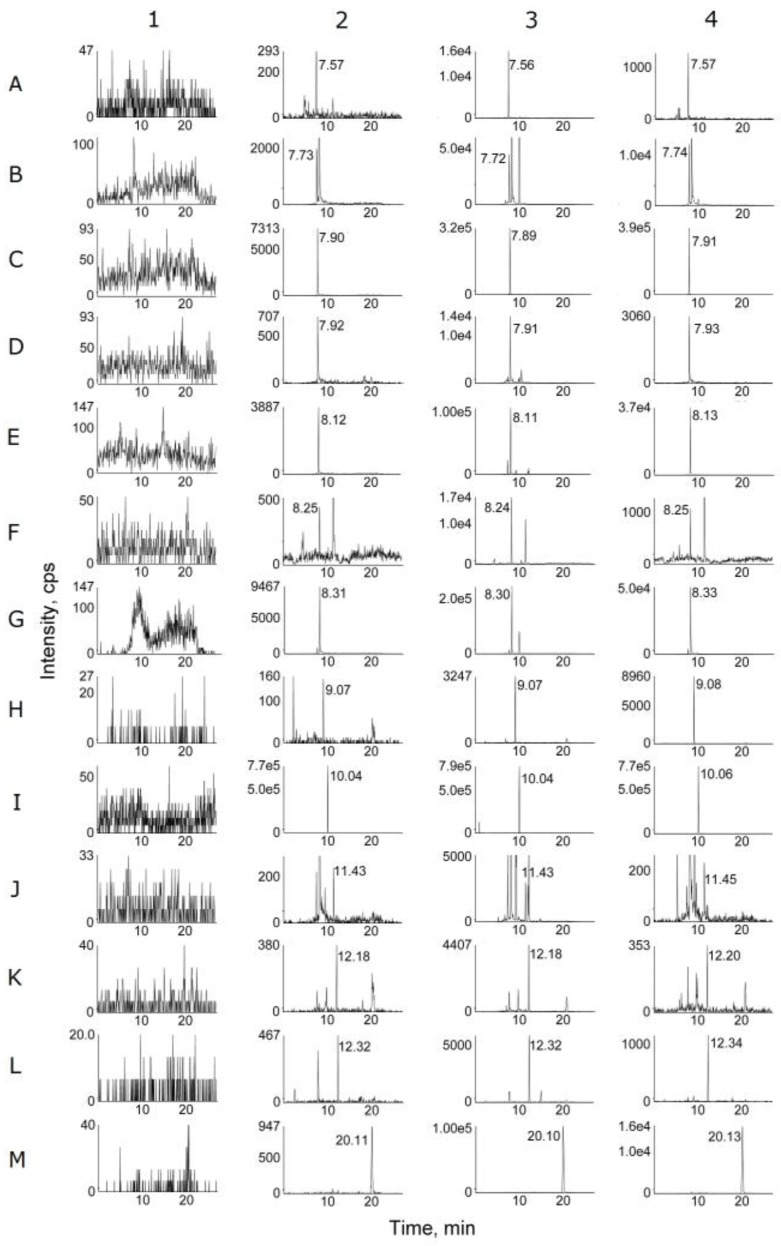
MRM chromatograms of blank plasma (**1**); plasma spiked with the 12 alkaloids at LLOQ (**2**); plasma samples at 1 h after oral administration of ZJ (**3**) and FZJ (**4**) extract. A (corydaldine), B (epiberberine), C (dehydroevodiamine), D (coptisine), E (palmatine), F (noroxyhydrastinine), G (berberine), H (wuchuyuamide-I), I (carbamazepine), J (8-oxoepiberberine), K (8-oxocoptisine), L (evodiamine), M (evocarpine).

Typical multiple reactions monitoring (MRM) mode chromatograms of blank plasma, plasma spiked with the 12 alkaloids at the lower limit of quantification (LLOQ) and IS, and plasma samples after oral administration of ZJ and FZJ extract were shown in Figure 2. Retention times (R_t_) were 7.57 (corydaldine), 7.73 (epiberberine), 7.90 (dehydroevodiamine), 7.92 (coptisine), 8.12 (palmatine), 8.25 (noroxyhydrastinine), 8.31 (berberine), 9.07 (wuchuyuamide-I), 10.04 (carbamazepine), 11.43 (8-oxoepiberberine), 12.18 (8-oxocoptisine), 12.32 (evodiamine), and 20.11 (evocarpine) min, respectively. There were no endogenous interferences in the MRM analysis of the alkaloids and IS in blank plasma.

Calibration curves of the 12 alkaloids had good linearity, and the regression equations, linear ranges, correlation coefficients and LLOQ are listed in Table S1. It should be noted that because the concentration of the analytes has been quadrupled during the sample preparation process, the actual lowest drug concentration determined in plasma was a quarter of the LLOQ. The intra-day and inter-day precisions (relative standard deviation, RSD) and accuracies (RE) of quality control (QC) samples were showed in Table S2. The precisions were less than 15% and accuracies were within ±15%. The limitation ranges were 20% and ±20% respectively for QC samples of low concentration. The results indicated that the method was reliable and reproducible for the analysis.

Absolute recoveries of the 12 alkaloids were all above 69% (Table S2). The results of matrix effect were listed in Table S2, which showed a slight ion suppression effect for four QPAs (from 78.16% to 85.16%), and an ion enhancement effect for wuchuyuamide-I (from 116.32% to 117.64%). However, no significant matrix effect was observed for IS (from 96.47% to 105.22%), and the RSD values were within the acceptable criteria, which indicated that there were no severe variations in the analysis. Stability results were presented in Table S3, which demonstrated that the 12 alkaloids were stable in rat plasma under different storage conditions and during sample processing.

### 2.4. PK Studies

**Figure 3:**
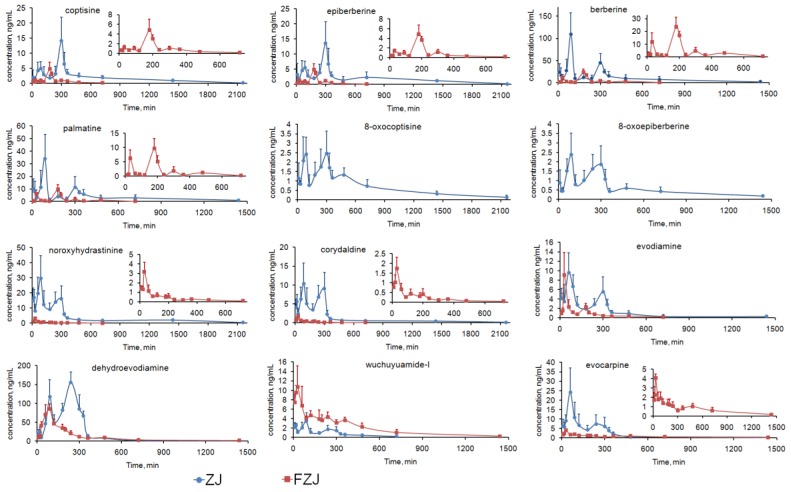
C–T curves of the 12 alkaloids after oral administration of ZJ and FZJ extract (*n* = 6).

The validated analytical method was successfully applied to the PK study of the 12 alkaloids after oral administration of ZJ and FZJ extract. The C–T curves of the 12 alkaloids were shown in Figure 3, and the corresponding PK parameters were listed in Table 1. Because double peaks were observed in the C–T curves of most of the alkaloids, observed maximum concentration and corresponding time values were used as C_max_ and T_max_. After oral administration of FZJ extract, plasma concentrations of 8-oxocoptisine and 8-oxoepiberberine at most time points were below the LLOQ, therefore the PK parameters couldn’t be calculated.

In drug PK studies, the C–T curve may reveal qualitative characteristics of PK profiles, while PK parameters such as AUC, MRT, C_max_ and t_1/2_, can reflect the systemic exposure level of the drug quantitatively. The PK results of the alkaloids of ZJ and FZJ were consequently discussed from the above two aspects.

#### 2.4.1. C–T Curves of the Alkaloids

Double peaks in C–T curves were observed repeatedly in the PK study of ZJ and related TCM prescriptions, but explanations to the phenomenon were still ambiguous. Generally, possible reasons for the double peak phenomenon could be summarized as follows: (a) enterohepatic circulation [36,37]; (b) two different sites of drug absorption [38,39,40]; (c) irregular pattern of gastric emptying [41,42,43,44]. In the present study, however, the factors contribute to double peaks of QPAs and the other alkaloids may be different.

(1) For the alkaloids except QPAs, double peaks were visibly observed in the C–T curves after oral administration of ZJ extract, but the secondary peaks were attenuated and the C–T curves approximately matched the two-compartment model after administration of FZJ (calculated by two fitting methods using DAS 2.0 software, Table 2). Moreover, T_max_ of the alkaloids after administration of ZJ extract were deferred from that of FZJ administration. Alkaloids of CR, especially berberine, have been reported to have inhibition effect on GI motility [45,46,47,48,49]. It probably play a major role in the double peak phenomenon, since the GI motility can affect the drug absorption. The content of berberine in ZJ extract were 20 times more than that in FZJ extract (Table S4), leading to more significant influence on the GI motility after oral administration. Thus, the double peak phenomenon and the delayed T_max_ of the alkaloids after administration of ZJ extract could be attributed to the inhibition effect on GI motility caused by CR alkaloids, especially berberine.
(2) In ZJ group, two plasma concentration peaks were observed at 90 and 300 min for all the four QPAs; but the primary plasma concentration peak was at 300 min for coptisine and epiberberine, and 90 min for palmatine and berberine (Table 1, significant differences between the plasma concentration values of the primary and secondary peaks were examined with *t*-test by Microsoft Office Excel 2007). Previous researches had suggested that the primary elimination route of berberine in vivo was renal excretion [50], and its C–T curve matched the two-compartment model following an intravenous administration [50,51]. Therefore, the enterohepatic circulation couldn’t be the major reason of the double peak phenomenon of QPAs, since the plasma concentration values of coptisine and epiberberine at 300 min were greater than that at 90 min. In addition, research findings of the main phase I metabolism of berberine [52,53,54] certified that metabolism couldn’t be the reason of the double peak phenomenon of QPAs. The absorption rate constant (Ka) of berberine and palmatine at jejunum had been reported to be greater than that at ileum and colon, which means the upper part of the intestine was their dominant absorption site [55,56,57]. Coptisine, in contrast, had a better absorption rate at colon compared with jejunum [57]. Therefore two absorption sites with different Ka could give a reasonable explanation about the double peak phenomenon of the four QPAs: plasma concentration peak at 90 min was mainly caused by the absorption at the upper part of the intestine, but mainly by the absorption at ileum and colon while that is at 300 min. Plasma concentration peaks of QPAs in FZJ group at 30 and 180 min were earlier than that of ZJ group, and peaks at 30 min caused by the absorption at the upper part of the intestine were attenuated. The possible reason was that the lower berberine content in FZJ weakened the inhibition effect on GI motility and subsequently led to the decreased residence time and absorption level of QPAs at upper part of the intestine.

#### 2.4.2. Systemic Exposure of the Alkaloids

Because the alkaloids content in ZJ and FZJ were no longer linear with the proportion of CR and EF after extraction process [58,59], AUC_0→∞_/D (ratio of AUC_0→∞_ and dose) and C_max_/D (ratio of C_max_ and dose) were calculated to estimate the dose modified systemic exposure levels of the 12 alkaloids (Table 3). MRT_0→∞_ and t_1/2_ values suggested that the 12 alkaloids were not eliminated rapidly in vivo, which indicated that absorptions were the major obstacle to their systemic exposure level. Physicochemical properties of these alkaloids had been calculated in silico to predict their human colon adenocarcinoma cell (Caco-2) permeability according to the three-property based rule (3PRule) [60]. Polar surface area (PSA), logarithm of the *n*-octanol/water distribution coefficient (LogD), molecular weight (MW), hydrogen bond donors (HBD), hydrogen bond acceptors (HBA), number of free rotatable bonds (NRB) and molar solubility (S) of the alkaloids were retrieved from SciFinder^®^ (American Chemical Society). Data of the in silico study were listed in Table S5. Physicochemical data of the four QPAs and dehydroevodiamine were not given because of the electric charge in their structures which hindered the calculation.

(1) The four QPAs from CR, especially berberine, had relatively high systemic exposure levels, but their AUC_0→∞_/D and C_max_/D values were extremely low. Previous studies suggested that absolute bioavailability of berberine was less than 1% [61,62], and it should be mainly attributed to its poor absorption. Transport experiments had confirmed that berberine, palmatine and coptisine had poor permeability across Caco-2 cell monolayer with apparent permeability coefficient (*P*_app_) values between 0.1 and 1.0 × 10^−6^ cm/s [63] as poorly absorbed compounds [64]. TPAs and SIAs, in contrast, were components with low contents in CR but higher AUC_0→∞_/D and C_max_/D values indicating their better absorption properties. 3PRule suggested that the two TPAs and two SIAs had favorable physicochemical properties for Caco-2 permeability: PSA ≤ 60, MW ≤ 400 and LogD > −1. However, compounds must be dissolved in water before penetrating the intestinal epithelial cells, and the suitable values of solubility (LogS) ranged from 0 to −4 [65]. Therefore, systemic exposure levels of two TPAs, especially 8-oxocoptisine, appeared to be limited by their lower solubility than SIAs.
(2) Evodiamine and dehydroevodiamine were two major IQAs in EF, but the results suggested that dehydroevodiamine had a higher systemic exposure level regardless of the dose modification. Our previous study demonstrated that both evodiamine and dehydroevodiamine had high permeability across Caco-2 cell monolayer with *P*_app_ values of 2.32 × 10^−5^ and 1.26 × 10^−5^ cm/s, respectively [66], which were close to the values predicted by 3PRule; but it was also found that the feeding concentration of evodiamine was limited due to its poor solubility in the transport experiment. Thus, its solubility should be the major obstacle to a higher systemic exposure level for evodiamine (predicted LogS < −5), and the poor solubility can be attributed to its flat, rigid, and unsaturated structure. In contrast, the solubilities of dehydroevodiamine and wuchuyuamide-I were improved because of the inner salt structure or the broken ring structure.
(3) Effects on the absorption and elimination of the alkaloids from CR and EF were illustrated by comparing the AUC_0→∞_/D, C_max_/D and t_1/2_ of the alkaloids after the administrations of ZJ and FZJ extract. Since the t_1/2_ values of some alkaloids, such as evodiamine, were not accurately calculated because of the multi-peak phenomenon, MRT_0→∞_ would be more reliable in the comparison. MRT_0→∞_ and t_1/2_ values of the four QPAs halved of FZJ group compared with that of ZJ group, but C_max_/D and AUC_0→∞_/D increased 4–9 times and 3–4 times, respectively. The comparisons indicated that absorptions of QPAs were increased, but their eliminations were accelerated as a result of increased EF intake. The QPAs like coptisine, palmatine and berberine were *P*-gp substrates [63,67], and their efflux ratio could be reduced by EF on the Caco-2 transport model [68], so EF may possibly promote the absorption of QPAs by inhibiting *P*-gp. On the other hand, EF could also induce hepatic UDP-glucuronosyltransferase 1A1 and then accelerate the elimination of QPAs [69]. In the present study, QPAs’ systemic exposure (AUC_0→∞_/D) were finally increased when the proportion of EF increased, suggesting that EF had a greater influence on the absorption than on the elimination of QPAs after the compatibility of CR and EF.

Contrary to QPAs, noroxyhydrastinine, corydaldine, dehydroevodiamine and evocarpine had a larger AUC_0→∞_/D and C_max_/D values in ZJ group than that in FZJ group. As argued above, high content of berberine in ZJ extract could cause inhibition effect on GI motility; and drug absorption could be subsequently improved when the GI motility was slowed probably because of the longer residence time [70,71,72], which might lead to the increased absorptions and systemic exposure levels of noroxyhydrastinine, corydaldine, dehydroevodiamine.

#### 2.4.3. Integrated PK Analysis

**Figure 4:**
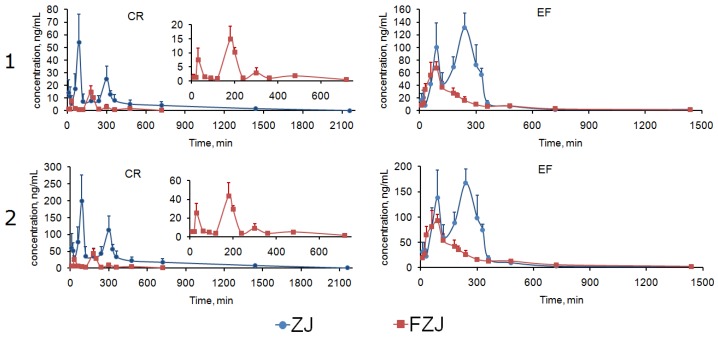
Integrated C–T curves of CR and EF alkaloids calculated by AUC-based weighting method (**1**) and total drug concentration method (**2**) after oral administration of ZJ and FZJ extract.

Among the 12 alkaloids, berberine and dehydroevodiamine had the most similar C–T curve profiles with that of the integrated C–T curves of CR and EF alkaloids (Figure 3 and Figure 4), indicating that berberine and dehydroevodiamine could be regarded as the representative components to reflect the PK behaviors of CR and EF alkaloids. The highest systemic exposure levels of berberine and dehydroevodiamine among the alkaloids, i.e., the biggest W_i_ values (Table 3), may contribute to this result. However, the AUC_0→∞_/D and C_max_/D values of berberine were extremely low, indicating that large quantity of berberine was not absorbed. It has been reported that at least 56% of berberine existed as prototype during the period of 0–36 h after intragastric administration, and there was 24% of berberine still remained in the GI tract even at 36 h [62]. In recent years, ZJ has been investigated for its antitumor activities, and its in vitro and in vivo antitumor activities against digestive system cancer have already been confirmed [2,3,4,5]. In addition, our previous studies showed that berberine, coptisine, and evodiamine had the inhibitory activities against the proliferation of human gastric carcinoma (NCI-N87) and Caco-2 cells with the half inhibitory concentration of 12.61–91.18 μmol/L, suggesting that they were probably the main effective components in ZJ against the digestive system cancer [9]. To epithelial tumor cells of digestive tract, oral drug could take effect directly without the absorption process, though the unabsorbed drug has been considered as useless traditionally. As for berberine, coptisine, and evodiamine, poor absorption would be conductive to their local effects in digestive tract; besides, GI retention effect caused by CR alkaloids could extend the treatment duration of these alkaloids. Therefore this research may give a support to the anti-digestive system cancer potentials of ZJ in the PK respect.

The two integrated methods had similar C–T curves profiles. However, the integrated AUC_0→∞_ and C_max_ values of CR and EF alkaloids calculated by AUC-based weighting method were significantly lower than total drug concentration method (examined using *t*-test by Microsoft Office Excel 2007, Table 4), and even lower than the values of berberine and dehydroevodiamine before integrated. Therefore the total drug concentration method was more concise and credible than the AUC-based weighting method to reflect the systemic exposure levels of CR and EF alkaloids.

## 3. Experimental Section

### 3.1. Chemicals and Materials

The rhizomes of *Coptis chinensis* Franch. (CR) were collected from “The National GAP Base of Chinese Materia Medica for Coptidis Rhizoma” at Shizhu County (Chongqing, China). The nearly ripe fruits of *Euodia rutaecarpa* (Juss.) Benth. (EF) were collected from Xiangtan city (Hunan, China). The two herbs were identified by Prof. Xiu-Wei Yang from School of Pharmaceutical Sciences, Peking University, and the voucher specimens have been deposited in School of Pharmaceutical Sciences, Peking University (Beijing, China).

Reference standards of the 12 alkaloids (coptisine, epiberberine, palmatine, berberine, 8-oxocoptisine, 8-oxoepiberberine, noroxyhydrastinine, corydaldine, evodiamine, evocarpine, dehydroevodiamine, and wuchuyuamide-I) were isolated and purified from ZJ [7,9] and EF [29,73], and their purities were more than 99%. Internal standard (IS) carbamazepine was acquired from National Institutes for Food and Drug Control (Beijing, China).

ACN and MeOH were LC–MS grade (J.T. Baker, Philipsburg, PA, USA). LC–MS grade formic acid was purchased from Dikma Technologies Inc. (Beijng, China), and LC–MS grade ammonium formate was purchased from Sigma-Aldrich Co. (St. Louis, MO, USA). Pure water (H_2_O) used in the analysis was prepared by Milli-Q system (Millipore Corporation, Billerica, MA, USA). Heparin sodium injection was purchased from Tianjin Biochem Pharmaceutical Co., LTD. (Tianjin, China). Other reagents were analytical grade and purchased from Beijing Chemical Works (Beijing, China).

### 3.2. Animals

The animal study was approved by the Animal Care and Use Committee of Peking University (approval No.: LA 2014161). Male Sprague-Dawley rats (weighed 220–250 g) were purchased from the Laboratory Animal Center of the Peking University Health Science Center (Beijing, China). The rats were housed with free access to laboratory food and water for 5 days, and deprived of food for 12 h before the experiment. The animal studies were carried out in accordance with the guidelines defined by the above approval on Animal Care and Use for the use of experimental animals.

### 3.3. Apparatus and Analytical Conditions

Analyses were performed on a DIONEX Ultimate 3000 HPLC system (Dionex Corp., Sunnyvale, CA, USA) and a 4000 triple quadrupole-linear ion trap mass spectrometer equipped with a Turbo VTM electrospray ionization (ESI) source (ABI/MDS Sciex, Concord, ON, Canada). Data acquisition and procession were performed with Analyst 1.5.1 software (ABI/MDS Sciex).

Chromatographic separation was achieved on a Diamonsil^®^ ODS-C_18_ column (250 mm × 4.6 mm, 5 µm, Dikma Technologies Inc., Beijng, China) at 25 °C. The mobile phase consisted of ACN (A) and H_2_O (B, containing 0.1% formic acid (*v*/*v*) and 11 mmol/L ammonium formate) with gradient elution program as follows: 10%→50% A at 0→6 min; 50%→80% A at 6→9 min; 80%→95% A at 9→13 min; 95% A at 13→20 min; 95%→10% A at 20→21 min; 10% A at 21→27 min. The flow rate was 1 mL/min and the sample injection volume was 5 µL.

The MS analysis was run in the multiple reactions monitoring (MRM) mode in the positive ionization. The MRM parameters of the 12 alkaloids and IS, including precursor ion (Q1), product ion (Q3), declustering potential (DP) and collision energy (CE) values were optimized (Table 5). The ion spray voltage was 4500 V and source temperature was 550 °C. Entrance potential and collision cell exit potential (CXP) were 10 V and 15 V, respectively. The nebulizing gas, auxiliary gas, curtain gas and collision activation dissociation gas were set at 50, 45, 20 and 2 psi with high-purity nitrogen.

### 3.4. Preparations of ZJ and FZJ Extracts

The extraction procedure of ZJ was based on the corresponding process of ZJ capsule in Pharmacopoeia of the People’s Republic of China [74]. The crushed CR (420 g) and EF (70 g) were mixed and refluxed 3 times with 10 times (*w*/*v*) of 60% aqueous ethanol for 1 h per time. The extracts were mixed and concentrated under reduced pressure to 0.6 g/mL crude drug, then lyophilized to afford ZJ extract (138.1 g, yield 28.2%). The crushed CR (70 g) and EF (420 g) were mixed and then extracted as described above to afford FZJ extract (153.5 g, yield 31.3%). The contents of the 12 alkaloids in ZJ and FZJ extracts were quantitatively determined (shown in Supplementary Table S4).

### 3.5. Preparation of Standard Solutions, Calibration Standards and Quality-Control Samples

The twelve accurately weighed alkaloids were dissolved in MeOH separately to prepare stock solutions. Stock solutions were mixed and serially diluted with MeOH to obtain working solution A (containing 0.56–720 ng/mL of coptisine, 0.54–700 ng/mL of epiberberine, 1.67–2160 ng/mL of palmatine, 3.70–4800 ng/mL of berberine, 0.35–450 ng/mL of 8-oxocoptisine, 0.39–510 ng/mL of 8-oxoepiberberine, 0.84–1090 ng/mL of noroxyhydrastinine, and 10.03–1300 ng/mL of dehydroevodiamine), and working solution B (containing 0.31–660 ng/mL of corydaldine, 0.44–960 ng/mL of evodiamine, 0.58–1260 ng/mL of wuchuyuamide-I, and 1.47–3180 ng/mL of evocarpine). The stock solution of IS was diluted to a concentration of 2040 ng/mL with MeOH as working solution. All the stock solutions were stored at 4 °C before use.

Mixed calibration standards of the 12 alkaloids were prepared by adding working solution A (30 μL) and B (30 μL) into 800 μL blank rat plasma. QC samples were prepared at low, medium and high concentrations in the same way.

### 3.6. Sample Preparation

Eight hundred μL of plasma samples (or calibration standards and QC samples) were spiked with 20 μL of IS solution (2040 ng/mL) and 4 mL of MeOH–ACN (2:1, *v*/*v*). The mixture was vortexed for 30 s, ultrasonic extracted for 2 min and centrifuged at 12,000 rpm for 10 min. The supernatant was transferred out and evaporated to dryness under a flow of gentle nitrogen gas at 45 °C. The residue was processed again with 600 μL of MeOH–ACN (2:1, *v*/*v*) as described above. Then the residue was resolved with 200 μL of MeOH–ACN (2:1, *v*/*v*), ultrasonic extracted for 2 min, and centrifuged at 12,000 rpm for 10 min. The supernatant was drawn out, and 5 μL of it was injected into the LC–MS/MS system for analysis.

### 3.7. Method Validation

The method was validated according to the guidance for bioanalytical method validation [75].

The specificity and selectivity was evaluated by comparing chromatograms of blank plasma from six rats, blank plasma spiked with the 12 alkaloids and IS, and plasma samples obtained from PK studies.

The lower limit of quantification (LLOQ) was defined as the lowest drug concentration on the calibration curve with signal-to-noise ratio (*S*/*N*) of 10:1, determined with precision (expressed as RSD) below 20% and accuracy (expressed as relative error, RE) within ±20%. The calibration curves for each of the 12 alkaloids were analyzed individually by fitting the peak area ratio response for analyte/IS as a function of standard concentration, using least square linear regression with a weighted factor (1/*x*^2^ for evocarpine, and 1/*x* for the other alkaloids). Accuracy and precision were evaluated by determining the QC samples at three concentration levels in six replicates on the same day and on three consecutive days.

The absolute recoveries of the 12 alkaloids were defined by comparing the peak areas of analytes in QC samples with those of the pure standard solutions containing equivalent amounts of the compounds. The matrix effects were evaluated by comparing the peak areas of the analytes dissolved with blank matrix extracts with those of pure standard solutions.

The stabilities of analytes in plasma were assessed by analyzing the QC samples at three concentration levels under the following storage conditions: after three freeze (−20 °C)-thaw (room temperature) cycles; stored at −20 °C for 30 days, and comparing the results with those of freshly prepared QC samples. Stabilities of the processed samples at room temperature for 24 h were also assessed.

### 3.8. Pharmacokinetic Study

The time schedule included 15 time-points and six rats were sampled at each time-point. All the rats were orally administered with ZJ extract (suspended in water) at a dose of 3.38 g/kg body weight (dosages of the 12 alkaloids were shown in Table S4), equivalent to crude drug dose of 12 g/kg body weight. Blood samples were collected from the suborbital vein of the rats at 10, 20, 30, 60, 90, 120, 180, 240, 300, 330, 360, 480, 720, 1440, 2160 min after administration, and centrifuged immediately at 5000 rpm for 10 min. The plasma samples were stored at −20 °C until analysis.

Similarly, the time schedule of other rats included 15 time-points and six rats were orally administered with FZJ extract at a dose of 3.76 g/kg body weight (also equivalent to crude drug dose of 12 g/kg body weight, Table S4). Blood samples were collected and treated as mentioned above in the PK study of ZJ extract.

PK parameters of the 12 alkaloids including AUC_0→t_, AUC_0→∞_, mean retention time (MRT_0→t_, MRT_0→∞_), and elimination half-life (t_1/2_) were calculated with non-compartmental method by DAS 2.0 software (Mathematical Pharmacology Professional Committee of China, Shanghai, China). Maximum plasma drug concentration (C_max_) and time to reach the maximum plasma drug concentration (T_max_) were observed directly from the detected C–T data. The results were expressed as arithmetic mean ± standard deviation (SD).

### 3.9. Integrated PK Analysis

#### 3.9.1. AUC-based Weighting Method

This method was based on the idea that the components of same type could be integrated as one component in the PK studies using AUC as the weighted factor [33]. For the major components of alkaloids of ZJ and FZJ, the structural types, properties and activities of alkaloids belonging to CR and EF were different, so they were integrated respectively. The weighted factors (W_i_) for alkaloids (expressed by “i”) of CR or EF were calculated with Formula (1). ΣAUC_0→∞_ represented the sum of the AUC_0→∞_ values of coptisine, epiberberine, palmatine, berberine, 8-oxocoptisine, 8-oxoepiberberine, noroxyhydrastinine and corydaldine for CR; as well as that of dehydroevodiamine, evodiamine, wuchuyuamide-I and evocarpine for EF. The integrated plasma concentrations (C_T_) of alkaloids of CR or EF were then calculated by Formula (2), where C_i_ represented the plasma concentration of each alkaloids of CR or EF acquired from the experiment. C_T_ of alkaloids of CR or EF was finally used to calculate the PK parameters in accordance with the procedure mentioned in Section 3.8.

(1) $$w_{i}=\frac{{\mathrm{AUC}}_{0\to \infty i}}{\sum {\mathrm{AUC}}_{0\to \infty }}$$

C_T_ = ΣC_i_ × W_i_ (2)

#### 3.9.2. Total Drug Concentration Method

In this method, alkaloids belonging to CR and EF were also integrated respectively. The integrated concentrations were summed up from the plasma concentrations of alkaloids of CR or EF without any weighted factors, and used to calculate the PK parameters in accordance with the procedure mentioned in Section 3.8.

## 4. Conclusions

Unlike other traditional herbal therapies across the world, TCM’s most important characteristic is multi-herb therapy based on its unique theory and treatment pattern [76]. Therefore pharmacokinetic studies of TCM formulae are more complicated because of the interactions of many kinds of components from the herbs; accordingly to investigate and reveal the interactions is a significant part of the pharmacokinetic research. In the present study, a sensitive LC–MS/MS method for simultaneous determinations of coptisine, epiberberine, palmatine, berberine, 8-oxocoptisine, 8-oxoepiberberine, noroxyhydrastinine, corydaldine, dehydroevodiamine, evodiamine, wuchuyuamide-I, and evocarpine in rat plasma has been developed. The PK behaviors of the 12 alkaloids after oral administration of ZJ and FZJ extracts were compared and the result was carefully discussed and analyzed. To sum up, the absorption, elimination and systemic exposure level of these alkaloids were mainly influenced by three factors: the proportion of EF and CR, the pharmacological effect on GI motility, and the physicochemical property of these alkaloids. In the practice, compatibility of EF and CR could well improve oral bioavailability of CR alkaloids, such as berberine. These new findings would be helpful for a better understanding of the activities and clinical applications of ZJ, FZJ and other related TCM prescriptions.

## Figures and Tables

●: C–T curves of the alkaloids after oral administration of ZJ. ■: C–T curves of the alkaloids after oral administration of FZJ.

**Table 1:** PK parameters of the 12 alkaloids in rat plasma after oral administration of ZJ and FZJ extract (mean ± SD, *n* = 6).

Analytes	Group	AUC_0→t_ (ng·h/mL)	AUC_0→∞_ (ng·h/mL)	MRT_0__→t_ (min)	MRT_0__→∞_ (min)	t_1/2_ (min)	T_max_ (min)	C_max_ (ng/mL)	T_sec_ (min)	C_sec_ (ng/mL)
coptisine	ZJ	68.73 ± 10.82	71.59 ± 10.72	641.65 ± 62.14	735.50 ± 91.37	480.47 ± 115.11	300	14.13 ± 7.75	90	4.96 ± 2.29 *
	FZJ	10.99 ± 1.64	11.85 ± 1.25	250.52 ± 27.45	312.29 ± 83.18	176.05 ± 73.47	180	4.78 ± 2.31	-	-
epiberberine	ZJ	68.46 ± 15.94	69.61 ± 15.46	645.43 ± 62.71	696.78 ± 87.57	366.42 ± 101.94	300	13.65 ± 7.05	90	5.51 ± 2.17 *
	FZJ	10.13 ± 1.09	11.40 ± 1.27	233.44 ± 25.91	315.37 ± 52.51	235.17 ± 101.63	180	4.82 ± 1.97	-	-
palmatine	ZJ	88.34 ± 24.92	96.63 ± 33.22	404.26 ± 105.70	525.01 ± 192.01	339.21 ± 144.61	90	34.07 ± 19.25	300	11.18 ± 8.71 *
	FZJ	18.19 ± 4.98	18.90 ± 5.50	231.35 ± 21.68	269.46 ± 38.99	150.78 ± 103.54	180	9.74 ± 3.53	30	6.25 ± 2.89 *
berberine	ZJ	277.48 ± 50.15	299.84 ± 55.27	387.56 ± 67.86	514.76 ± 164.64	378.28 ± 194.53	90	109.40 ± 48.27	300	44.68 ± 21.28 *
	FZJ	48.07 ± 9.60	50.24 ± 10.74	244.37 ± 17.40	280.76 ± 37.56	133.26 ± 50.10	180	23.67 ± 7.42	30	11.64 ± 7.28 *
8-oxocoptisine	ZJ	24.74 ± 3.90	27.02 ± 2.23	607.27 ± 59.56	809.87 ± 254.49	535.67 ± 158.71	300	2.46 ± 1.17	90	2.42 ± 0.89
	FZJ	-	-	-	-	-	-	-	-	-
8-oxoepiberberine	ZJ	14.30 ± 2.32	16.63 ± 3.22	450.77 ± 59.37	740.15 ± 138.92	592.65 ± 274.26	90	2.38 ± 1.14	300	1.85 ± 0.99
	FZJ	-	-	-	-	-	-	-	-	-
noroxyhydrastinine	ZJ	133.19 ± 18.49	140.06 ± 17.83	529.35 ± 75.19	662.05 ± 153.25	644.66 ± 169.16	90	29.64 ± 15.15	300	16.29 ± 8.38
	FZJ	4.95 ± 0.65	5.37 ± 0.73	181.73 ± 16.42	314.79 ± 172.07	243.81 ± 175.97	30	3.17 ± 1.00	-	-
corydaldine	ZJ	45.14 ± 7.46	47.27 ± 7.36	402.95 ± 56.32	513.14 ± 109.81	595.01 ± 123.69	90	10.34 ± 5.45	300	9.03 ± 4.31
	FZJ	2.86 ± 0.35	3.10 ± 0.46	170.94 ± 11.39	239.52 ± 47.43	233.20 ± 77.36	30	1.73 ± 0.58	-	-
dehydroevodiamine	ZJ	532.34 ± 57.78	537.43 ± 54.97	260.43 ± 15.40	282.90 ± 34.14	192.57 ± 135.94	240	155.16 ± 27.92	90	117.29 ± 45.45
	FZJ	274.77 ± 23.19	285.60 ± 24.08	279.59 ± 56.33	357.86 ± 114.71	301.96 ± 172.11	90	85.27 ± 13.37	-	-
evodiamine	ZJ	31.71 ± 2.94	31.84 ± 3.11	315.19 ± 44.82	359.80 ± 45.66	141.31 ± 71.63	60	9.59 ± 4.22	300	5.54 ± 3.14
	FZJ	10.83 ± 1.77	11.72 ± 2.06	178.31 ± 22.86	254.67 ± 42.11	227.88 ± 62.98	30	9.10 ± 4.79	180	2.21 ± 0.85
wuchuyuamide-I	ZJ	11.54 ± 1.40	12.77 ± 1.43	231.61 ± 27.74	313.20 ± 68.30	218.14 ± 89.78	90	3.45 ± 1.73	20	2.55 ± 0.38
	FZJ	48.25 ± 8.86	50.11 ± 8.81	346.41 ± 46.61	413.10 ± 94.85	295.01 ± 109.89	30	10.73 ± 4.43	-	-
evocarpine	ZJ	55.65 ± 12.40	59.02 ± 13.34	251.37 ± 29.26	390.99 ± 119.47	539.67 ± 383.48	60	24.20 ± 12.95	240	7.36 ± 4.89
	FZJ	17.84 ± 3.99	19.57 ± 4.79	422.27 ± 69.79	557.88 ± 117.71	414.88 ± 60.07	30	4.07 ± 0.44	-	-

T_sec_ and C_sec_ were the time and drug concentration of the secondary peak in C–T curve. * *p* < 0.05 compared with C_max_.

**Table 2:** Two-compartment model fitting result of the alkaloids in rat plasma after oral administration of ZJ and FZJ extract.

Analytes	ZJ		FZJ	
	*r*^2^	AIC	*r*^2^	AIC
noroxyhydrastinine	0.313 ± 0.350	118.137 ± 17.623	0.802 ± 0.128	16.505 ± 12.486
corydaldine	0.243 ± 0.267	85.431 ± 9.718	0.805 ± 0.066	3.947 ± 8.187
dehydroevodiamine	0.324 ± 0.280	154.46 ± 11.416	0.886 ± 0.055	107.718 ± 6.140
evodiamine	0.428 ± 0.169	71.547 ± 6.327	0.853 ± 0.232	30.067 ± 6.112
wuchuyuamide-I	0.544 ± 0.140	35.057 ± 11.851	0.519 ± 0.407	66.844 ± 7.076
evocarpine	0.734 ± 0.201	78.232 ± 11.393	0.740 ± 0.167	27.61 ± 10.527

*r*^2^: correlation coefficient method; AIC: Akaike’s information criterion method.

**Table 3:** Dose modified AUC_0→∞_, C_max_ and AUC weighted factors (W_i_) of the 12 alkaloids in rat plasma after oral administration of ZJ and FZJ extract.

Analytes		ZJ			FZJ		
		AUC_0→∞_/D	C_max_/D	W_i_ *	AUC_0→∞_/D	C_max_/D	W_i_ *
CR alkaloids	coptisine	0.40	0.08	0.09	1.80	0.72	0.12
	epiberberine	0.62	0.12	0.09	1.91	0.81	0.11
	palmatine	0.77	0.27	0.13	3.03	1.56	0.19
	berberine	0.84	0.31	0.39	2.56	1.21	0.50
	8-oxocoptisine	44.44	3.98	0.04	-	-	-
	8-oxoepiberberine	97.25	13.92	0.02	-	-	-
	noroxyhydrastinine	1687.47	357.11	0.18	268.50	158.50	0.05
	corydaldine	1688.21	369.29	0.06	310.00	173.00	0.03
EF alkaloids	dehydroevodiamine	39.69	11.46	0.84	12.15	3.63	0.78
	evodiamine	8.55	2.57	0.05	3.73	2.89	0.03
	wuchuyuamide-I	172.57	46.62	0.02	278.39	59.61	0.14
	evocarpine	35.17	14.42	0.09	13.16	2.74	0.05

D denoted the dosage of the 12 alkaloids, see Table S4; * Calculated by AUC-based weighting method.

**Table 4:** Integrated PK parameters of the CR and EF alkaloids in rat plasma after oral administration of ZJ and FZJ extract (mean ± SD, *n* = 6).

PK Parameters	Integrated Method	ZJ		FZJ	
		CR Alkaloids	EF Alkaloids	CR Alkaloids	EF Alkaloids
AUC_0→t_ (ng·h/mL)	A	165.85 ± 25.24	454.02 ± 49.50	31.01 ± 4.38	222.33 ± 18.87
	B	740.58 ± 96.29	632.55 ± 69.91	97.24 ± 12.03	352.74 ± 31.92
AUC_0→∞_ (ng·h/mL)	A	174.90 ± 23.60	458.50 ± 46.98	36.92 ± 8.61	230.95 ± 19.29
	B	790.50 ± 129.01 *	640.61 ± 64.83 *	113.78 ± 19.69 *	368.20 ± 33.08 *
MRT_0→t_ (min)	A	465.48 ± 62.56	260.53 ± 15.54	251.24 ± 19.62	282.29 ± 55.06
	B	498.47 ± 54.24	262.85 ± 17.19	243.73 ± 17.92	295.12 ± 49.32
MRT_0→∞_ (min)	A	464.99 ± 65.15	283.73 ± 34.85	254.95 ± 60.94	359.90 ± 111.27
	B	513.57 ± 53.75	293.62 ± 40.77	272.09 ± 72.64	373.48 ± 90.75
t_1/2_ (min)	A	537.02 ± 408.47	195.02 ± 136.85	141.62 ± 31.75	303.14 ± 162.88
	B	573.75 ± 447.62	218.36 ± 137.70	186.77 ± 91.91	334.42 ± 104.19
T_max_ (min)	A	90	240	180	90
	B	90	240	180	90
C_max_ (ng/mL)	A	54.14 ± 22.14	131.18 ± 23.45	14.82 ± 4.70	67.02 ± 10.36
	B	198.70 ± 76.91 *	167.10 ± 28.40 *	43.79 ± 14.12 *	92.367 ± 12.51 *
Tsec (min)	A	300	90	30	-
	B	300	90	30	-
Csec (ng/mL)	A	24.99 ± 10.08	99.90 ± 38.75	7.51 ± 4.19	-
	B	113.27 ± 40.70	138.18 ± 54.42	25.35 ± 10.26	-

A denoted the AUC-based weighting method, and B denoted the total drug concentration method. * *p* < 0.05 compared with method A.

**Table 5:** MRM parameters of the 12 alkaloids and IS.

Analytes	Q1 (Da)	Q3 (Da)	DP (V)	CE (eV)
coptisine	320.1	292.1	110.1	42.3
epiberberine	336.1	320.2	101.0	46.2
palmatine	352.1	336.3	86.9	42.7
berberine	336.1	292.1	85.7	46.8
8-oxocoptisine	336.1	308.2	102.6	38.1
8-oxoepiberberine	352.2	308.3	105.5	48.2
noroxyhydrastinine	192.2	119.1	74.6	33.8
corydaldine	208.2	165.1	68.3	26.3
dehydroevodiamine	301.7	286.2	99.6	53.0
evodiamine	304.2	134.2	91.8	36.4
wuchuyuamide-I	352.3	158.2	55.8	31.7
evocarpine	340.2	186.1	136.5	54.5
carbamazepine (IS)	237.1	194.2	76.9	25.0

## Supplementary Materials

The following are available online at http://www.mdpi.com/1420-3049/22/2/214/s1. Table S1: Summary of regression equations, linear ranges, correlation coefficients and LLOQ of the 12 alkaloids in rat plasma, Table S2: Summary of the precisions, accuracies, recoveries and matrix effect, Table S3: Stability of the 12 alkaloids in rat plasma under different storage conditions (*n* = 6), Table S4: Contentsand dosagesof the 12 alkaloids in ZJ and FZJ extract, Table S5: Calculated physicochemical properties and predicted Caco-2 permeability of the alkaloids.
