# Supplementary material for: Pharmacokinetics Studies of 12 Alkaloids in Rat Plasma after Oral Administration of Zuojin and Fan-Zuojin Formulas
Source: Molecules. 2017 Jan 30;22(2):214. doi: 10.3390/molecules22020214 (PMC6155683; doi:10.3390/molecules22020214)
Supplement: Supplementary file 1 [file molecules-22-00214-s001.pdf]

Supplementary Materials: Pharmacokinetics Studies of 12 Alkaloids in Rat Plasma after Oral Administration of Zuojin and Fan-Zuojin Formulas

Ping Qian, You-Bo Zhang, Yan-Fang Yang, Wei Xu and Xiu-Wei Yang

Table S1. Summary of regression equations, linear ranges, correlation coefficients and LLOQ of the 12 alkaloids in rat plasma.

Analytes	Regression Equations	Correlation Coefficients (r)	Linear Ranges (ng/mL)	LLOQ (n = 6)		
				LLOQ (ng/mL)	RSD (%)	RE (%)
coptisine	Y = 0.00127X + 0.00182	0.9992	108.00–0.25	0.25	15.92	−9.51
epiberberine	Y = 0.00422X + 0.00477	0.9978	105.00–0.24	0.24	15.88	−16.20
palmatine	Y = 0.00468X + 0.02126	0.9988	324.00–0.75	0.75	17.13	−19.29
berberine	Y = 0.00352X + 0.04189	0.9984	720.00–1.67	1.67	15.89	−2.59
8-oxocoptisine	Y = 0.00101X + 0.00033	0.9985	67.50–0.16	0.16	10.58	13.57
8-oxoepiberberine	Y = 0.00115X + 0.00008	0.9976	76.50–0.18	0.18	11.87	−13.33
noroxyhydrastinine	Y = 0.00034X + 0.00009	0.9955	163.50–0.38	0.38	10.92	−13.03
corydaldine	Y = 0.00088X + 0.00014	0.9963	99.00–0.14	0.14	18.95	−6.01
dehydroevodiamine	Y = 0.00245X + 0.00685	0.9941	975.00–1.50	1.50	7.68	−10.21
evodiamine	Y = 0.00036X + 0.00012	0.9988	189.00–0.26	0.26	19.45	5.17
wuchuyuamide-I	Y = 0.00084X + 0.00008	0.9973	144.00–0.20	0.20	12.37	−9.08
evocarpine	Y = 0.00544X + 0.00086	0.9904	477.00–0.66	0.66	13.39	−19.55

Table S2. Summary of the precisions, accuracies, recoveries and matrix effect of the 12 alkaloids in rat plasma (n = 6).

Analytes	Concentration (ng/mL)	Intra-Day		Inter-Day		Recovery (%)		Matrix Effect (%)	
		RSD (%)	RE (%)	RSD (%)	RE (%)	mean	RSD	mean	RSD
coptisine	54.00	14.34	−8.40	13.07	−8.10	71.66	11.52	81.34	13.37
	4.50	12.40	−0.74	11.66	−4.88	71.19	11.29	78.16	11.09
	0.25	15.92	−9.51	15.02	−9.68	84.57	3.40	83.70	16.54
epiberberine	52.50	8.92	−5.37	9.30	−8.52	75.80	9.47	80.90	14.01
	4.38	9.84	−13.45	10.97	−11.44	77.49	12.22	80.48	5.88
	0.24	15.88	−16.20	19.31	0.67	81.96	5.81	83.56	9.21
palmatine	162.00	8.67	−9.26	9.79	−9.55	82.77	11.23	83.99	10.97
	13.50	13.84	−4.09	13.05	−6.32	75.09	6.34	80.81	4.94
	0.75	17.13	−19.29	16.53	−15.21	98.42	3.97	85.16	7.71
berberine	360.00	9.19	−13.61	11.72	−12.39	78.14	14.04	82.82	10.51
	30.00	11.04	4.66	11.15	1.05	82.78	6.59	82.57	4.21
	1.67	15.89	−2.59	15.01	6.27	100.78	5.67	84.45	8.13
8-oxocoptisine	33.75	9.30	−7.46	12.39	2.00	71.91	13.02	86.58	13.78
	2.81	14.26	6.70	11.27	6.93	73.06	10.62	87.90	13.49
	0.16	10.58	13.57	18.61	9.35	83.90	5.39	90.33	17.08
8-oxoepiberberine	38.25	9.90	−9.67	8.10	−8.53	69.22	13.45	87.68	14.77
	3.19	12.77	−2.65	12.66	0.71	75.40	14.76	93.32	8.41
	0.18	11.87	−13.33	14.16	−9.89	103.53	10.32	95.07	11.30
noroxyhydrastinine	81.75	2.10	−9.97	6.47	−9.49	97.45	3.55	92.72	4.93
	6.81	6.71	−1.27	10.76	−0.82	89.62	7.50	99.22	3.57
	0.38	10.92	−13.03	12.31	−12.79	114.57	6.89	94.92	19.23
corydaldine	49.50	3.64	−7.81	6.60	−7.18	80.97	2.45	85.66	6.06
	6.19	6.52	−6.68	8.99	−8.74	88.66	9.93	89.03	1.68
	0.14	18.95	−6.01	15.52	3.06	101.01	8.57	107.03	15.88
dehydroevodiamine	975.00	12.23	3.42	12.58	1.70	75.54	9.94	82.71	6.54
	81.25	13.66	8.02	13.58	4.83	69.03	10.00	81.85	12.06
	4.51	13.92	7.89	18.50	−2.01	84.97	7.68	85.44	19.68
evodiamine	94.50	14.09	1.70	12.70	1.44	70.86	8.65	99.78	14.26
	11.81	11.23	−12.07	11.26	−10.37	79.92	8.88	98.67	16.23
	0.26	19.45	5.17	17.54	0.81	97.14	10.87	114.73	15.92

	72.00	4.77	−7.73	7.50	−7.37	119.18	7.50	117.64	3.30
wuchuyuamide-I	9.00	8.06	−13.34	6.08	−13.90	123.05	6.21	117.53	6.92
	0.20	12.37	−9.08	16.81	−0.21	114.31	9.90	116.32	17.16
	238.50	4.11	2.37	8.99	2.43	80.74	9.70	85.12	14.18
evocarpine	29.81	4.13	−8.64	11.58	−9.16	75.44	8.58	84.23	7.72
	0.66	13.39	−19.55	18.42	3.43	88.61	11.35	96.03	15.51

Table S3. Stability of the 12 alkaloids in rat plasma under different storage conditions (n = 6).

Analytes	Concentration (ng/mL)	3 Freeze-Thaw Cycles		30 Days at −20 °C		Processed Samples′ Stability	
		RSD (%)	RE (%)	RSD (%)	RE (%)	RSD (%)	RE (%)
	54.00	8.2	0.97	4.13	0.65	12.43	−8.19
coptisine	4.50	10.53	5.77	13.67	−8.99	7.99	−2.14
	0.25	19.26	6.92	17.65	−11.58	17.57	−10.47
	52.50	11.77	−12.84	6.81	−6.2	9.86	−10.77
epiberberine	4.38	10.09	−6.12	10.51	−13.86	11.02	−8.85
	0.24	19.1	−3.64	15.29	13.92	15.67	2.78
	162.00	8.81	−12.89	10.21	−12.35	9.15	−12.47
palmatine	13.50	14.48	−7.2	6.51	−13.85	13.84	−2.85
	0.75	15.2	−14.23	13.41	−17.74	18.48	−9.27
	360.00	11.6	−14.86	7.79	−14.65	10.36	−13.71
berberine	30.00	10.26	−1.36	12.29	−13.68	9.45	4.57
	1.67	18.54	7.87	18.7	−8.36	14.43	9.52
	33.75	11.41	−12.48	10.96	−14.51	13.41	10.05
8-oxocoptisine	2.81	11.85	7.48	13.25	9.66	7.59	10.68
	0.16	13.36	6.73	15.52	6.72	18.7	−3.71
	38.25	9.04	−8.93	13.09	−2.71	6.78	−7.73
8-oxoepiberberine	3.19	2.54	0.37	11.33	0.36	11.8	9.35
	0.18	10.45	−14.18	6.4	−13.41	14.53	−7.14
	81.75	5.21	−9.82	3.26	−12.1	5.55	−12.23
noroxyhydrastinine	6.81	4.24	11.57	7.34	−2.75	13.18	−0.19
	0.38	8.97	−14.7	17.23	−8.89	15.08	−8.14
	49.50	3.6	−2.29	4.17	−2.68	4.06	−10.59
corydaldine	6.19	6.53	3.89	8.75	−11.86	10.31	−6.31
	0.14	16.11	−1.98	17.12	15.65	7.86	13.85
	975.00	7.74	5.86	8.65	4.85	11.72	−1.09
dehydroevodiamine	81.25	11.78	14.45	14.59	−1.12	13.33	4.78
	4.51	6.43	4.36	8.65	−6.34	18.15	−0.76
	94.50	4.93	14.57	6.65	12.86	12.61	2.87
evodiamine	11.81	6.55	−2.19	11.32	−9.8	12.45	−6.55
	0.26	13.55	6.06	14.83	−6.27	17.75	1.14
	72.00	11.84	0.37	9	−1.38	6.71	−10.58
wuchuyuamide-I	9.00	9.47	−14.69	8.06	−10.79	4.52	−14.07
	0.20	11.56	18.91	14.12	14.46	7.16	15.1
	238.50	11.94	7.91	9.05	6.12	9.59	3.83
evocarpine	29.81	6.94	3.44	9.87	−14.67	14.23	−14.47
	0.66	18.34	−19.16	12.56	8.28	8.77	14.05

Table S4. Contents and dosages of the 12 alkaloids in ZJ and FZJ extract.

Analytes	ZJ Extract		FZJ Extract	
	Content (mg/g)	Dosage (mg/kg Body Weight)	Content (mg/g)	Dosage (mg/kg Body Weight)
coptisine	52.772	178.369	1.755	6.599
epiberberine	33.192	112.189	1.586	5.963
palmatine	37.164	125.614	1.660	6.242
berberine	105.275	355.830	5.210	19.590
8-oxocoptisine	0.180	0.608	0.004	0.015
8-oxoepiberberine	0.051	0.172	0.002	0.008
noroxyhydrastinine	0.025	0.085	0.005	0.019
corydaldine	0.008	0.027	0.003	0.011
dehydroevodiamine	4.006	13.540	6.253	23.511
evodiamine	1.102	3.725	0.837	3.147
wuchuyuamide-I	0.022	0.074	0.048	0.180
evocarpine	0.497	1.680	0.395	1.485

Table S5. Calculated physicochemical properties and predicted Caco-2 permeability of the alkaloids.

Analytes	PSA (Å^2)	LogD (pH = 7)	MW	HBA	HBD	RBN	LogS (pH = 7)	Permeability Class*	P_{app} * (10^{-6} cm/s)
8-oxocoptisine	57.2	4.65	335.3	6	0	0	−6.07	High	33.2
8-oxoepiberberine	57.2	2.64	351.4	6	0	2	−4.66	High	33.2
noroxyhydrastinine	47.6	0.75	191.2	4	1	0	−2.48	High	33.2
corydaldine	47.6	1.37	207.2	4	1	2	−2.21	High	33.2
evodiamine	39.3	3.89	303.4	4	1	0	−5.17	High	33.2
wuchuyuamide-I	90.0	0.97	351.4	7	2	4	−2.70	Moderate–High	9.8
evocarpine	20.3	5.75	339.5	2	0	11	−6.48	High	33.2

* Predicted Caco-2 permeability according to the three-property based rule (3PRule).
